# Rare Forms of Castleman Disease Mimicking Malignancy: Mesenteric and Pancreatic Involvement

**DOI:** 10.7759/cureus.2310

**Published:** 2018-03-12

**Authors:** Mustafa Ozsoy, Zehra Ozsoy, Suleyman Sahin, Yuksel Arıkan

**Affiliations:** 1 Department of General Surgery, Parkhayat Hospital, Afyon / Turkey; 2 Department of Internal Medicine, State Hospital, Afyon / Turkey; 3 Department of General Surgery, Faculty of Medicine, Afyon Kocatepe University, Afyon / Turkey

**Keywords:** castleman’s disease, lymphoproliferative disorder, abdominal localization, peripancreatic tumor

## Abstract

Castleman disease is a lymphoproliferative disorder with unknown etiology and pathogenesis. While the disease may involve all parts of the body, the mediastinum appears to be the most common part of involvement. In this study, we present two cases of Castleman disease with different localizations that mimicked malignancy. A 62-year-old female patient presented with jaundice. Laboratory analysis indicated aspartate aminotransferase: 250 U/L, total bilirubin: 4 mg/dl, and carbohydrate antigen (CA) 19-9: 900 U/ml. Computerized tomography (CT) of the abdomen showed a mass originating from the pancreas head which resulted in a biliary tract obstruction. A positron emission tomography-computed tomography (PET/CT) showed that the only site of involvement was the pancreas head. A decision was made to perform pancreaticoduodenectomy. During intra-abdominal exploration, lymphadenopathies were identified in the surroundings of the retropancreatic portal vein and the hepatic artery. Histopathological investigation of the dissected lymph nodes demonstrated findings consistent with granulomatous plasma-cell-rich Castleman disease. A 55-year-old female patient presented with abdominal pain, nausea, and vomiting. Computerized tomography of the abdomen showed an abdominal mass of 7 cm, originating from the mesenterium, with high-contrast uptake in the mesenterium in the lower abdominal quadrant. The mesenteric mass was resected along with segmentary small intestine resection. Histopathological investigation of the mass showed a giant granulomatous structure that consisted of plasma cells consistent with Castleman disease. Castleman disease should be kept in mind during differential diagnosis of locally advanced lymph nodes observed during preoperative investigations and intraoperative exploration.

## Introduction

Castleman disease is a lymphoproliferative disease that was first described in 1956 by Castleman [1]. While the disease may occur in any part of the body, mediastinum is the most common (70%) site of involvement. Other frequent sites of involvement include the axilla, pelvis, and retroperitoneal regions. Based on the extent of clinical involvement, it is classified into two sub-types: unicentric and multicentric. The most commonly seen type is unicentric Castleman, which often follows an asymptomatic course [2]. In this study, we present two different cases of Castleman disease that mimicked malignancy.

## Case presentation

Case 1

**Figure 1:**
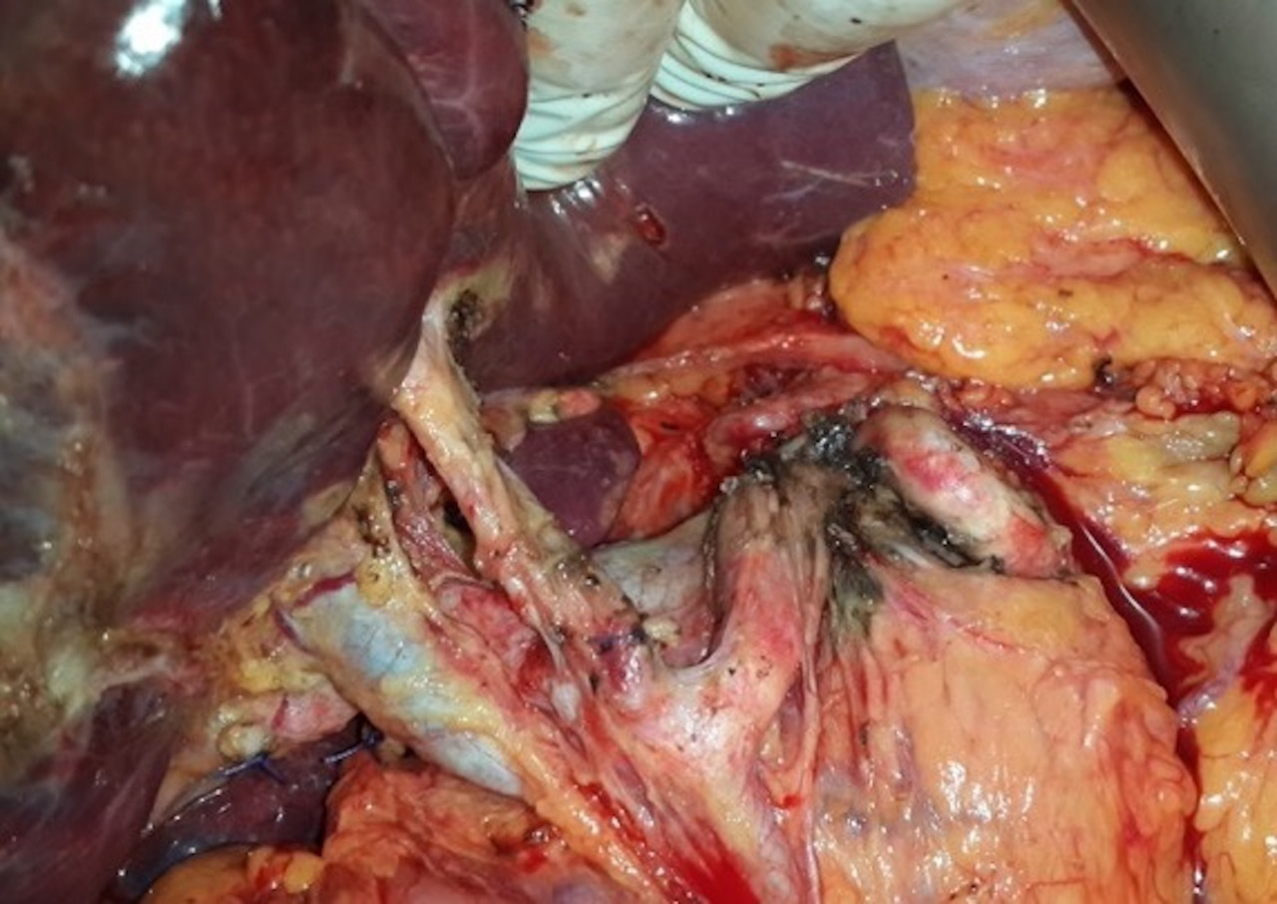
Intraoperative appearance after hepatoduodenal and celiac lymph node dissection

A 62-year-old female patient presented with jaundice. Over the previous month, the patient had complaints of nausea and vomiting, which increased after meals. The patient’s medical and family history did not include any specific characteristics. Her laboratory results were as follows; aspartate aminotransferase (AST): 250 U/L, total bilirubin: 4 mg/dl, and carbohydrate antigen (CA) 19-9: 900 U/ml. Computerized tomography (CT) of the abdomen showed a mass of approximately 4 cm, which originated from the pancreas head and resulted in a biliary tract obstruction. A positron emission tomography-computed tomography (PET/CT) showed that the only site of involvement was the pancreas head. A decision was made to perform a pancreaticoduodenectomy. No mass was seen in the pancreas head when the duodenum was ventilated by expanded Kocher maneuver during intra-abdominal exploration. Lymphadenopathies were noted surrounding the retro-pancreatic portal vein and the hepatic artery. As similar findings were not detected in any other abdominal foci during intra-abdominal exploration, the celiac artery and the lymph nodes around the vena porta were dissected (Figure 1).

**Figure 2:**
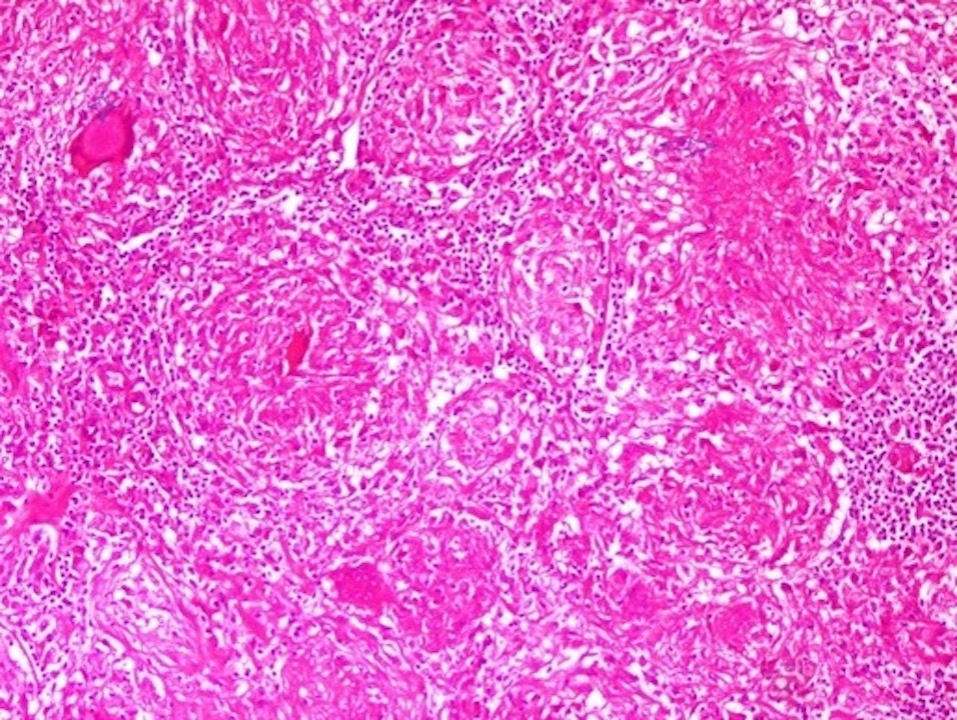
Granuloma structure consisting of giant plasma cells with central caseification necrosis (HEx100)

Histopathological investigation of the piece demonstrated findings consistent with Castleman disease rich in granulomatous plasma cells (Figure 2).

Case 2

**Figure 3:**
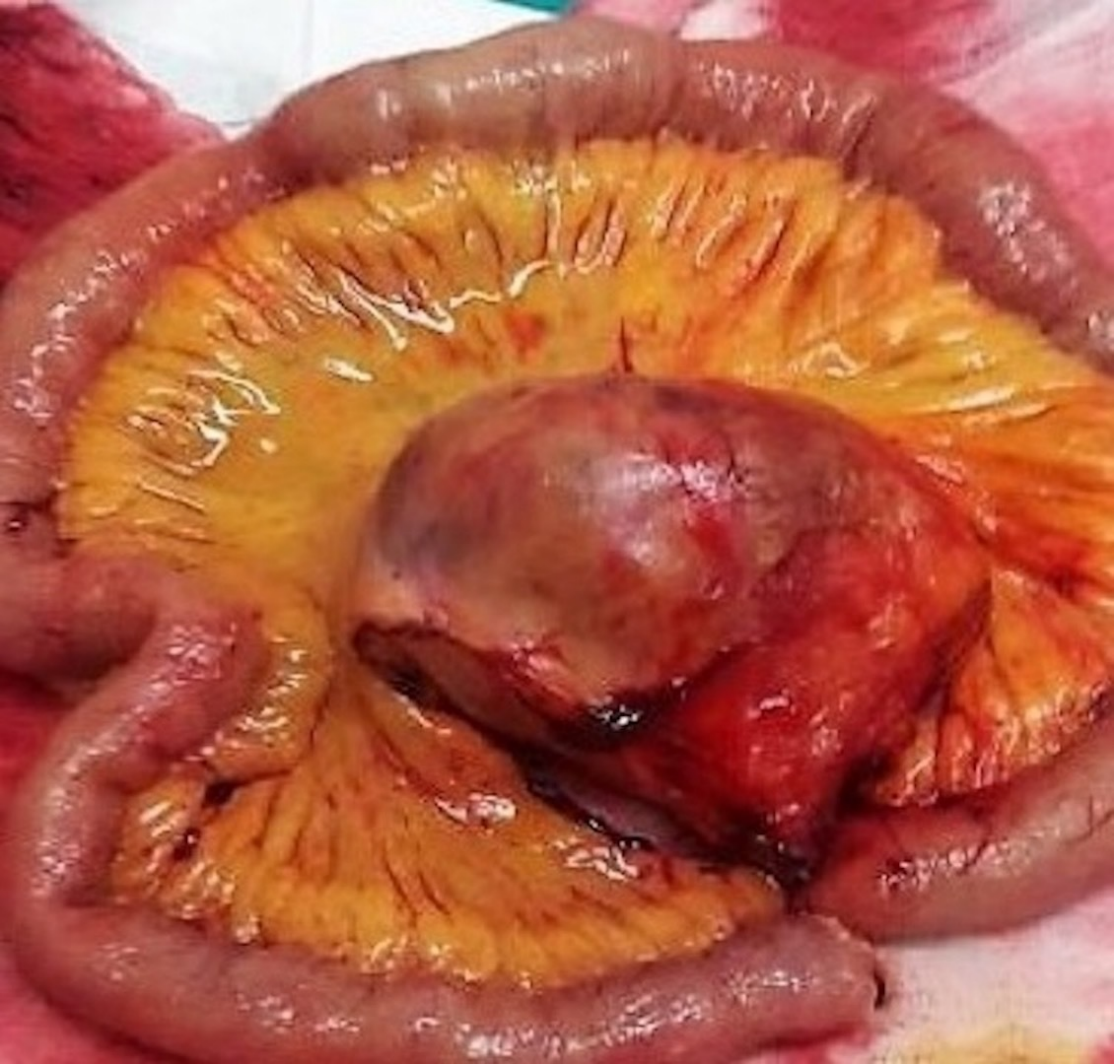
Mesenteric Castleman disease resected along with partial small intestine resection

A 55-year-old female patient presented with abdominal pain, nausea, and vomiting. The patient’s medical and family history did not include any specific characteristic. Her blood pressure was 140/80 mmHg, and pulse was 85 (min) at presentation. Laboratory analysis did not indicate any significant pathology. Computerized tomography of the abdomen showed an abdominal mass of mesenteric origin sized 6.7*6*5.5 cm, with intense contrast uptake in the mesenterium in the lower abdominal quadrant. The patient had no additional morbidity, and a decision was made for surgery. Intra-abdominal exploration revealed a mass with regular margins, localized at the mesenteric root at approximately 50 cm proximal of the terminal ileum. The mesenteric mass was resected together with segmentary small intestine resection (Figure 3).  

**Figure 4:**
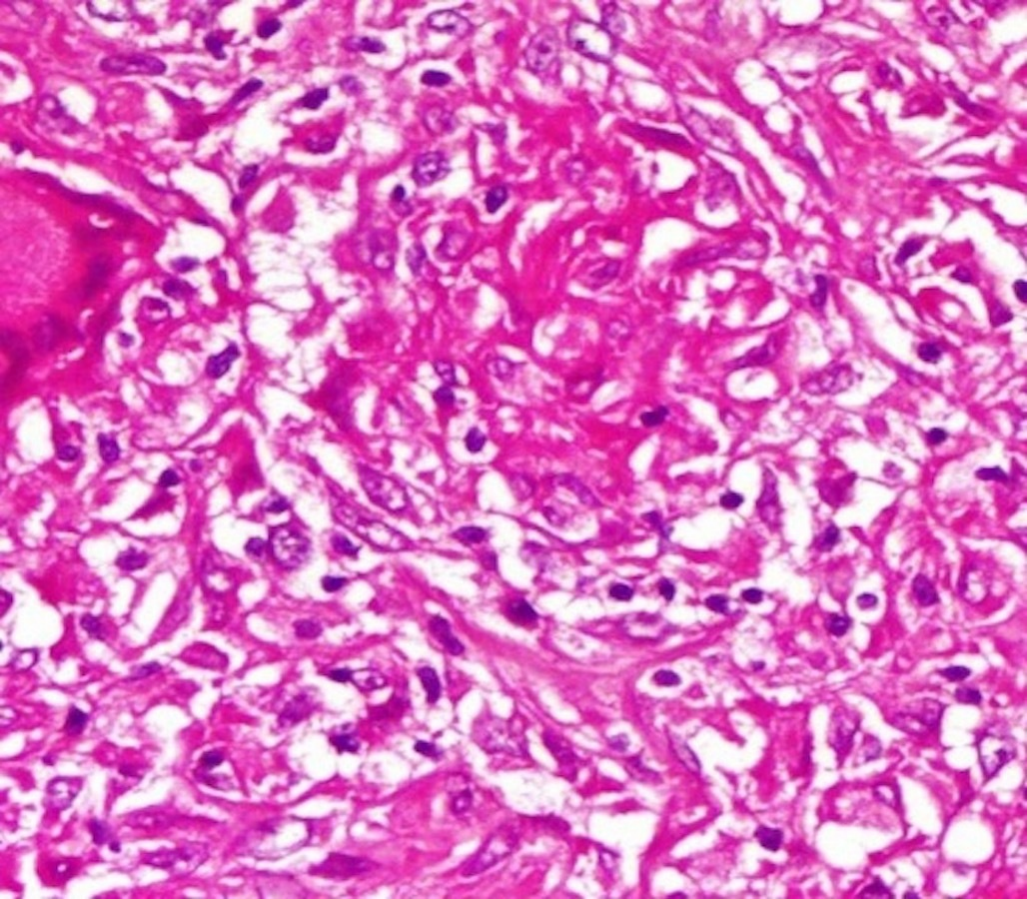
Granuloma structure rich in hyaline-vascular type plasma cells (HEx100)

Histopathological investigation of the mass revealed a giant granulomatous structure that consisted of plasma cells. In light of this information, the patient was screened for potential granulomatous diseases, such as tuberculosis and sarcoidosis. The patient was diagnosed with Castleman disease, as she was found to be negative for granulomatous diseases (Figure 4). The patient is currently in the postoperative twelfth month, and she had been followed-up without any complications.

## Discussion

Castleman disease was first defined by Castleman upon identifying enlarged lymph nodes that mimicked thymoma, in the mediastinum of 13 patients [1]. While the etiology and pathophysiology of Castleman disease are still unknown, potential contributors to disease development include chronic inflammation, immune deficiencies, and autoimmune diseases. Additionally, Epstein-Barr virus, Toxoplasma, and Mycobacterium tuberculosis are among the infectious agents that are held responsible for disease development. Clinical and laboratory abnormalities noted in the presence of Castleman disease are associated with inflammatory mediators, particularly with interleukin-6 [2-3]. The contribution of inflammatory mediators to disease development was clearly demonstrated in Komatsuda’s research [4].

CD is clinically classified into two types: unicentric and multicentric CD. Unicentric (90%) is the most commonly-seen type of CD. Multicentric type, which is a far less-frequent form of the disease, is associated with a poor prognosis. Patients with multicentric Castleman disease often die early due to infections and other causes secondary to malignancy. When a patient is diagnosed with multicentric CD, the disease should be considered as systemic, and combination treatment, including chemotherapy, steroids, and anti-IL-6, should be initiated as soon as possible [5]. The most frequently encountered form of CD is unicentric. In this variant, the disease is often incidentally detected. While the most-common clinical picture involves a slowly-growing asymptomatic mass, a small percentage (10%) of patients may experience symptoms such as fever, weight-loss, weakness, and symptoms associated with the pressure of the mass. Duration of symptoms varies from a few weeks to months. Based on its histopathological characteristics, Keller classified CD into two types as hyaline- and plasma-cell CD [2-5].

The most commonly-seen pathological form is the hyaline-vascular type. Histopathologically, the hyaline-vascular type consists of small lymphocytes, vascular stoma, and plasma cells clustered around multiple germinative centers in the form of a onion skin. While CD may involve almost every part of the body, abdominal involvement is very rare. Tuberculosis, lymphoma, accessory spleen, and the other hypervascular lesions should also be considered when CD is seen with abdominal involvement. Mesenteric involvement was previously defined in only 22 cases in the literature. While most of the cases were the hyaline-vascular type, they also involved potentially resectable masses [6]. Contrary to the previously-defined cases in the literature, our patients had a symptomatic disease that involved weight loss, abdominal pain, and other symptoms due to the pressure of the mass. While mesenteric involvement was unicentric, histopathological investigations indicated hyaline-vascular type CD. CD is difficult to diagnose preoperatively. The disease often presents with a solitary mass. Enlarged solitary lymph node showing homogeneous intense enhancement upon administration of contrast agent in computerized tomography should remind the diagnosis of the CD. The disease is frequently confused with malignancy as unicentric CD does not have specific radiological findings and appears as a solitary mass on radiological images.  There are also cases where a definite diagnosis could be made based on biopsy samples obtained preoperatively by endoscopic ultrasonography [7].

However, this technique is still inadequate due to limited implementation, and the low rate of accurate diagnosis. In both cases presented here, computerized tomography was the preferred diagnostic tool for preoperative investigations. In the study, Guo et al. described two cases of CD that mimicked pancreatic cancer [8]. Similar to the levels seen in the presence of pancreatic cancer, the cases had elevated levels of CA19-9. A definitive diagnosis of CD can be made postoperatively by investigating the specimens obtained during surgery. Additionally, intraoperative frozen investigations can guide the diagnosis.

The case presented here had lymphadenopathies in the hepatoduodenal region and retropancreatic area, which obstructed the biliary tract. Contrast enhancement in CT and PET/CT, weight loss, jaundice, and elevated CA19-9 levels initially lead to the diagnosis of pancreas malignancy. The disease can be treated with surgery, radiotherapy, steroids, and immunotherapy (interferon α and anti-IL-6 antibodies) or by a combination of them. Total resection is the most commonly preferred curative treatment option for the unicentric CD. Prognosis after total surgical resection is almost perfect [9]. Partial resection is also beneficial in cases where complete resection is not possible. The rate of recurrence is very low, even after partial resection.

Therefore, aggressive surgical treatment of CD is not recommended as this may increase the rates of morbidity and mortality among CD patients. CD is a benign disease, which may also be treated by chemoradiotherapy as an alternative to surgery or after surgery. Although there are conflicting results in the literature, it is commonly agreed that chemoradiotherapy is not a definitely curative therapeutic option. In the presence of unresectable unicentric CD, neoadjuvant rituximab and neoadjuvant radiotherapy can allow resection to be performed with a lower rate of morbidity given that these treatments may result in tumor shrinkage and reduced vascularity [10]. Total resection provided cure in both cases presented here.

## Conclusions

Castleman disease should be kept in mind during the differential diagnosis of locally advanced lymph nodes observed during preoperative investigations and intraoperative exploration.

Informed Consent: Written informed consent was obtained from patient who participated in these cases.

Conflict of Interest: No conflict of interest was declared by the authors.

Financial Disclosure: The authors declared that this study has received no financial support.

## References

[1] Castleman B, Iverson L, Menendez VP (1956). Localized mediastinal lymph node hyperplasia resembling thymoma. Cancer.

[2] Keller AR, Hochholzer L, Castleman B (1972). Hyaline-vascular and plasma-cell types of giant lymph node hyperplasia of the mediastinum and other locations. Cancer.

[3] Casper C (2005). The aetiology and management of Castleman disease at 50 years: translating pathophysiology to patient care. Br J Haematol.

[4] Komatsuda A, Wakui H, Togashi M, Sawada K (2010). IgA nephropathy associated with Castleman disease with cutaneous involvement. Am J Med Sci.

[5] Nishimoto N, Kanakura Y (2005). Humanized antiinterleukin- 6 receptor antibody treatment of multicentric Castleman disease. Blood.

[6] Greco LG, Tedeschi M, Stasolla S, Gentile A, Gentile A, Piscitelli D (2010). Abdominal nodal localization of Castleman's disease: Report of a case. Int J Surg.

[7] Khashab MA, Canto MI, Singh VK, Ali SZ, Fishman EK, Edil BH, Giday S (2011). A rare case of peripancreatic Castleman's disease diagnosed preoperatively by endoscopic ultrasound-guided fine needle aspiration. Endoscopy.

[8] Guo H, Shen Y (2012). Castleman disease mimicked pancreatic carcinoma: report of two cases. World J Surg Oncol.

[9] Chen CH, Liu HC, Tung KY, Lee JJ, Liu CL, Liu TP (2007). Surgical outcome of superficial and deep Castleman disease. ANZ J Surg.

[10] Baek HJ, Kook H, Han DK, Shin MG, Kim HS, Hwang TJ (2012). Unicentric Castleman disease relapsed after fituximab-CHOP chemotherapy or radiation therapy in an adolescent. J Pediatr Hematol Oncol.

